# Spatiotemporal characteristics of lower back muscle fatigue during a ten minutes endurance test at 50% upper body weight in healthy inactive, endurance, and strength trained subjects

**DOI:** 10.1371/journal.pone.0273856

**Published:** 2022-09-13

**Authors:** Christoph Anders, Tim Schönau

**Affiliations:** Division of Motor Research, Pathophysiology and Biomechanics, Experimental Trauma Surgery, Department for Hand, Reconstructive, and Trauma Surgery, Jena University Hospital, Friedrich-Schiller University Jena, Jena, Germany; University of Hartford College of Education Nursing and Health Professions, UNITED STATES

## Abstract

In modern developed societies, heavy physical demands are decreasing and getting replaced by longer periods of static, low-exertion activities such as sitting or standing. To counteract this lack of physical activity, more and more people are engaging in physical activity through exercise and training. Virtually opposite training modalities are endurance and strength. We asked if back muscle endurance capacity is influenced by training mode. 38 healthy male subjects (age range 19–31 years, mean age 22.6 years) were investigated: sedentary (Control, n = 12), endurance trained (ET, n = 13), and strength trained participants (ST, n = 13). They underwent a ten-minutes isometric extension task at 50% of their upper body weight. Surface EMG was measured in the low-back region utilizing quadratic 4*4 monopolar electrode montages per side. Relative amplitude and mean frequency changes were analysed with respect to electrode position and group during the endurance task. Eight ST subjects failed to complete the endurance task. Relative amplitude and frequency changes were largest in the ST group, followed by Control and ET groups (amplitude: F 6.389, p 0.004, frequency: F 11.741, p<0.001). Further, independent of group largest amplitude increase was observed for the most upper and laterally positioned electrodes. Mean frequency changes showed no systematic spatial distribution pattern. Although, in the light of an aging population, strength training has its merits our results question the functional suitability of frequent and isolated high-impact strength training for everyday endurance requirements like doing the dishes. Fatigue related amplitude elevations are systematically distributed in the back region, showing least fatigue signs for the most caudal and medial, i.e. the lumbar paravertebral region.

## 1 Introduction

Any repeated physical activity causes muscular fatigue if it exceeds critical duration and/or intensity [1, 2]. Muscular fatigue is a complex and reversible process that results in "any exercise-induced reduction in the ability of a muscle to generate force or power" [2]. It has different causes which can mainly be attributed to "a progressive reduction in voluntary activation of muscle during exercise", i.e. central fatigue and "fatigue produced by changes at or distal to the neuromuscular junction", i.e. peripheral fatigue [2]. This reduced force capacity leads to a relative increase of submaximal workload levels approaching this temporally reduced maximum force level, or in other words, reduce the physiological reserve during submaximal tasks. Considering the essential role of human abdominal and back muscles to ensure spinal stability, trunk muscle fatigue for any reason bears the risk of instability and thus stress on ligaments and the spine itself [3, 4]. If during such vulnerable situations loads act on the trunk at least subfailure injuries are to be expected [5], possibly leading to back pain with no detectable morphologic signs [5]. Because exercise-induced fatigue temporarily impairs adequate core muscle function, reduced exercise levels could have similar but permanent effects on core muscles.

In todays, highly developed countries, due to a more inactive, sedentary lifestyle a reduced physical performance is apparent in the majority of people [6]. This trend is negatively augmented by a sedentary life style in combination with high-calorie food intake [7], putting people in a vicious circle of reduced energy demands, degeneration of active tissues (i.e. muscles), elevated metabolic supply, overweight, and therefore further reduced physical activity. On the other hand, recreational sports activities are gaining more and more attention. People deliberately decide to increase their physical activity since they recognize the overall positive effects of physical training on physical [8] and mental [9] health. Furthermore, as an incomplete listing, physical activity has positive effects on the development of metabolic diseases like type II diabetes [10], decreases fracture susceptibility [11], and also results in a positive body image [12]. In addition, in the context of an increasingly aging population, any effort to postpone age-related involution with its effects on mobility also plays an essential role, for example for fall prevention [13] and social participation [14, 15].

As already stated, a sedentary life style also leads to deconditioning of the trunk muscles, which is discussed as one of possible causes for the development of acute and chronic back pain [16, 17] because the necessary spinal segmental stabilization is corrupted [18, 19]. If looking at rehabilitation or training programs trunk muscle training is often performed by using specific training devices that either apply isometric forces [20–22], or in case of dynamic exercises limit range and direction of motion to ensure a safe training in terms of preventing possible injuries [23]. However, all these devices apply an artificial training that may not be functionally adequate for everyday requirements. More functionally oriented trunk muscle training approaches like back school [24] and the spinal stabilizing training [25–27] aim at the involvement of adequate trunk stability and mobility to most effectively protect the spinal column from injury. Nevertheless, trunk muscles are involved in almost all physical activities. Therefore, more general than just specific trunk exercises should also have an impact on the trunk and especially the back muscles.

As todays physical demands decrease in general but involve more endurance than high load components [28, 29] we asked, if different training modalities i.e. specific endurance and strength training have impact on back muscle’s endurance capacity. Endurance-trained subjects should demonstrate superior endurance capacity and lower muscular fatigue in comparison with untrained healthy subjects. In the strength-trained subjects, we expected reduced endurance performance compared to endurance-trained, but also untrained subjects.

## 2 Methods

### 2.1 Participants

For this study, 38 healthy male participants were recruited by announcement and direct contact with the respective training groups. The investigated population consisted of one group of healthy subjects with only minor to normal physical activity serving as the control group (CON, n = 12) and two groups of physically active people. These two active groups were competing and practised either endurance (ET, cycling and triathlon, n = 13) or strength training (ST, power lifting, n = 13) at competition level (at least four training sessions per week, training duration four to 15 years). Both training regimes did not particularly target training of the back muscles. The comparatively low number of participants was due to a challenging recruitment of the two active groups. For reasons of equal group sizes the CON group was also limited to the same number of participants. All participants were informed about the procedure and aim of the study and signed informed consent to voluntarily participate in this investigation. To account for possible gender-related differences, only male subjects were investigated [30]. The study was approved by the ethics committee of the Friedrich-Schiller University Jena (2020-1844-BO). Inclusion criteria were either no sports at all (group CON, activity level 0 to 2, for details see S1 Text), or regular physical endurance or strength training at least four times per week (groups ET and ST, minimal activity level 4, for details see S1 Text). The age range of study participants was limited to 18 to 35 years (age range of the studied cohort: 19–31 years) to include only adult participants without no apparent age-related muscle regression [31]. To exclude relevant orthopedic and neurologic disorders, participants were clinically investigated and further queried about their medical history. Besides general health problems possibly interfering with unrestricted study participation, specific exclusion criteria were any surgery of the back and past or actual back pain. Details about the study participants are provided in Table 1.

**Table 1:** Participant characteristics and selected anthropometric data.

	Endurance	Strength	Control
**Activity level (1–5)**	5.0 ± 0	4.8 ± 0.4	2.3 ± 0.7^§^^, ^^$^ ^<0.0001^
**Age [years]**	22.2 ± 2.9	23.5 ± 1.8	22.1 ± 1.0
**Height [cm]**	184 ± 6.5	180 ± 5.6	184 ± 5.9
**Weight [kg]**	72.8 ±.7.0	90.3 ± 13.9 ^§^ ^0.003^	78.9 ±.14.3^$^ ^0.024^
**BMI [kg/m^2^]**	21.5 ± 1.3	27.7 ± 3.8 ^§^ ^<0.0001^	23.1 ±.3.8 ^$^ ^0.007^
**UBW [kg]**	31.7 ± 2.8	37.0 ± 4.5 ^§^ ^0.002^	32.7 ± 4.5
**UBT [Nm]**	123 ± 12.3	134 ± 21.8	125 ± 19.5

BMI: body mass index; UBW: upper body weight; UBT: upper body torque

§ indicates significant differences vs. endurance, $ indicates significant differences vs. strength, together with the respective significance levels

For definition of activity levels please see S1 Text

### 2.2 Investigation

**Fig 1:**
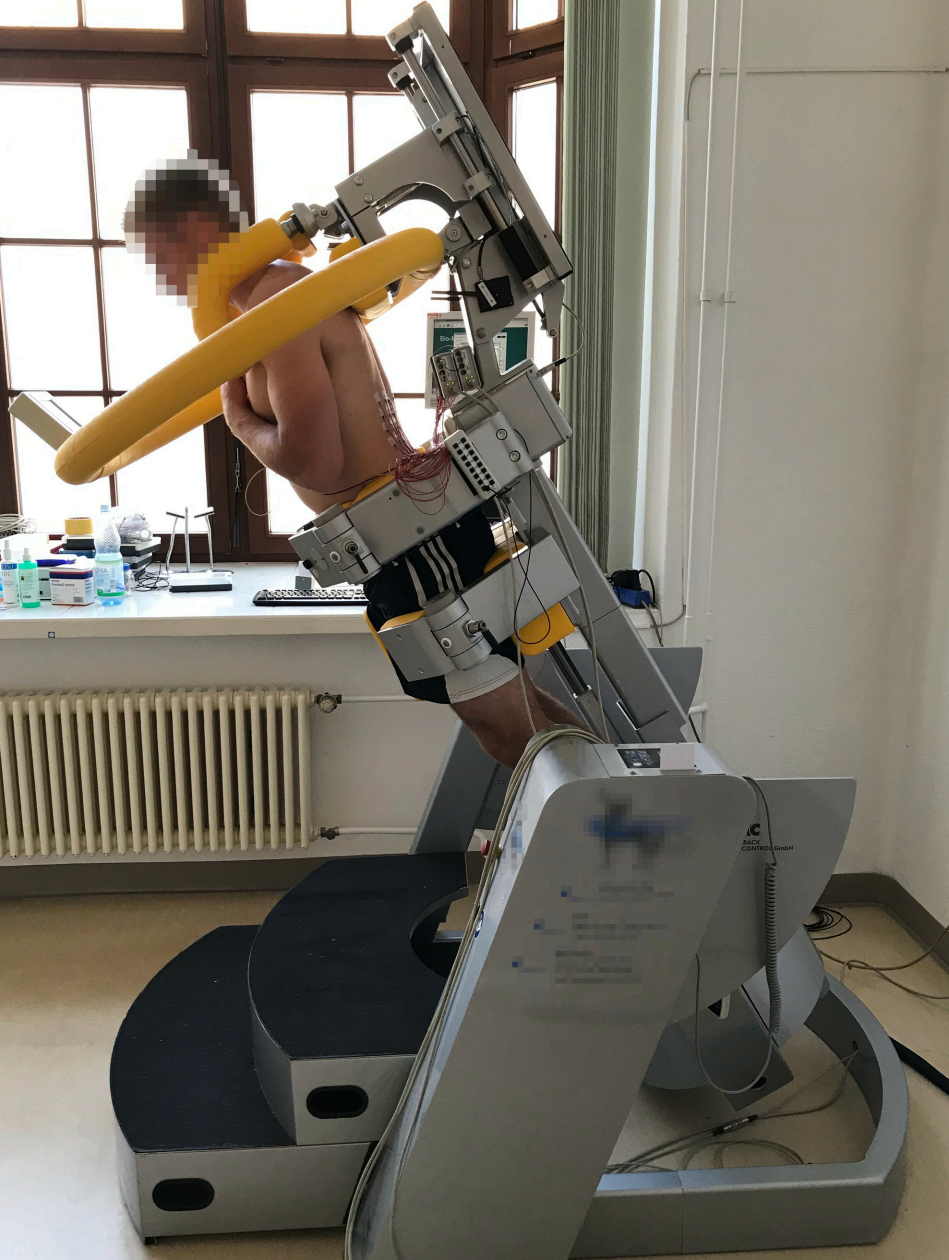
Example of a participant during the endurance test at 30° forward tilt. Please note the arms posture (crossed over chest) and the biofeedback monitor in front of the subject’s head.

Participants were positioned in a computerized test and training device for trunk muscles (CTT Centaur, BfMC, Germany) in upright standing position. In this device, the participant’s lower body is fixed while the upper body maintains limited freedom of movement (depending on the subject’s thoracic anthropometry a maximum of about 5 cm in anterior-posterior direction). The device is equipped with a harness, positioned over the subjects’ shoulder that contains strain gauges for force measurement in frontal and sagittal directions. By tilting the device forward until it is horizontal, gravitational forces act on the trunk (Fig 1), enabling the application of defined load levels up to 100% of the subject’s upper body weight (UBW) if tilted 90° (i.e. horizontal position). We determined the individual UBW while participants were tilted to horizontal position. In this position participants leaned relaxed in the harness. Residual back muscle contractions were determined by Surface EMG (SEMG, see below) and reported to the participants for correction. This was performed three times and the largest and trustworthy value was further used as the UBW (largest out of three values, not deviating more than 10%). UBT values were determined by correcting the UBW values by the distance between palpable L4 spinous process and the horizontal projection of the medial end of the scapular spine at the spine. For the endurance task, participants were exposed to 50% UBW extension load by 30° forward tilt. The subject’s task requirement was simply to maintain upright body position while being tilted forward. Correct adherence to upright body position was enabled by a small biofeedback monitor, located in front of the participants (Fig 1). In the biofeedback monitor a moving target point is displayed that is located in the centre of a crosshair if no net force acts on the harness, i.e. the subject maintains upright body posture. During the endurance task participants were asked to hold their arms crossed over their chests (see Fig 1).

For this endurance test 600 s exposure to 50% of UBW was defined as the target time, as this combination according to pre-investigations proved to be an adequate task requirement to induce relevant muscular fatigue without provoking exhaustion, i.e. to evoke muscular failure. Participants were requested to maintain their upright position until target time was reached, or until exhaustion.

### 2.3 EMG measurement and analysis

SEMG was measured monopolarly from the back region of both sides. At every side, electrodes were positioned in a 4*4 quadratic electrode arrangement that was adjusted to each participant’s anthropometry by defining the edge length of the electrode grid as the distance between palpable spines of L4 and L1 to cover the entire lumbar spine. We decided to adopt the grid to the participant’s anthropometry, to fully cover the lumbar region. According to the investigated population, this distance varied between seven and nine centimetres. For reliable electrode placement, we used prepared grid templates with the inner column of electrodes always placed 1.5 cm lateral to midline and the lower and upper rows at L4 and L1 vertebral positions, respectively. Electrodes were always applied by the same experienced investigator (TS).

**Fig 2:**
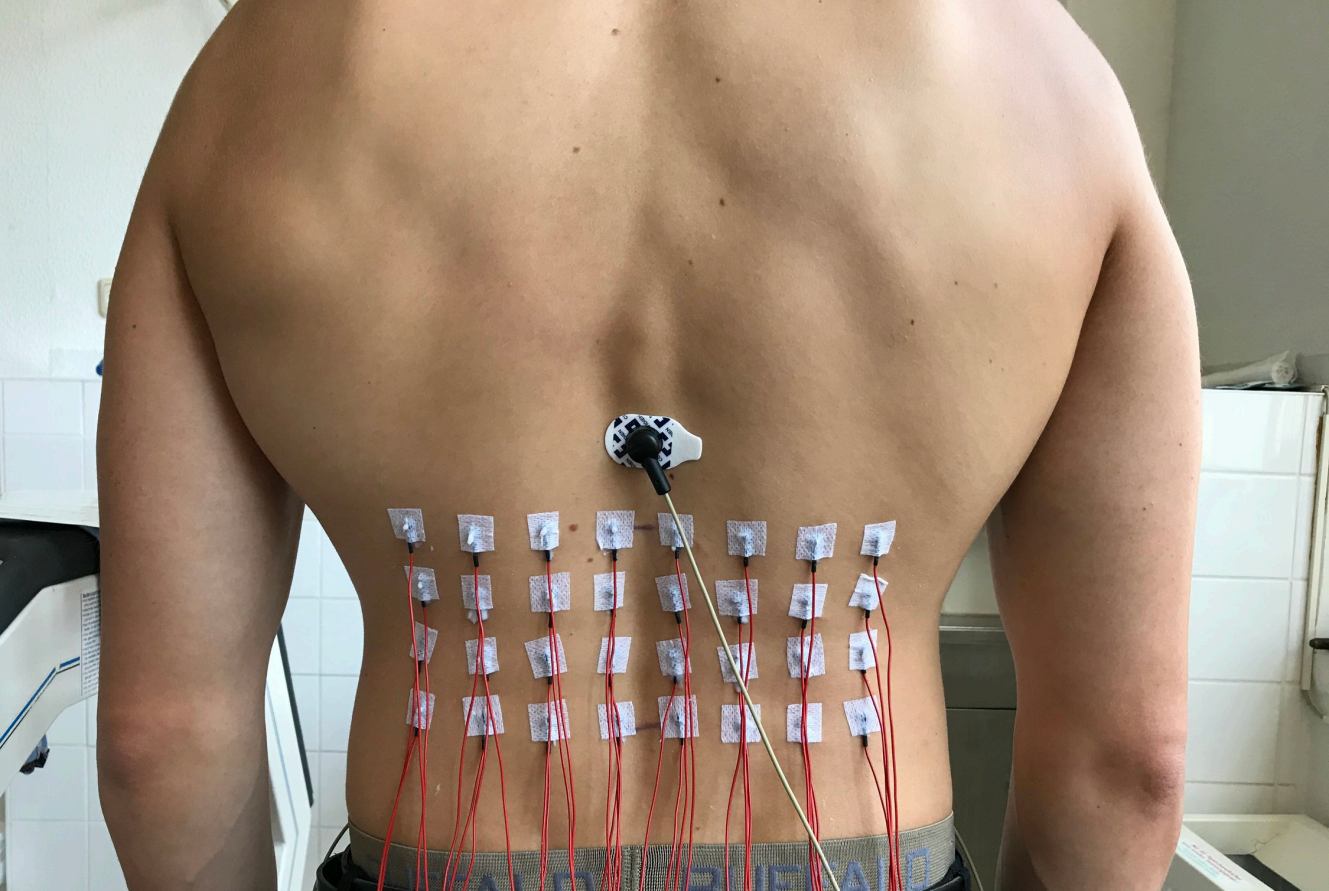
Aspect of the electrode arrangement. The separate electrode at Th11 level served as the ground electrode.

For this investigation reusable Ag-AgCl cup electrodes with a diameter of 6 mm and a hole at their apex for electrode gel application were used (DAGS102606, gvb geliMED, Germany). After gently cleaning, shaving, and rubbing (SkinPure, Nihon Kohden, Japan) the investigation region, electrodes were placed and fixed at the defined positions with elastic patches, containing a small hole that was positioned just at the electrodes apex for electrode gel application (Electrode Cream, Care Fusion, Finland; Fig 2). During electrode application, special attention was payed to the cables pointing downwards to avoid lever off the electrodes.

Electrodes were then connected to the monopolar amplifiers (ToEM16G: 10 - 1,861 Hz (-3 dB), gain: 100, input impedance: 22 MOhm, SNR: 1.13 μV_eff_, CMRR: 91.6 dB; DeMeTec, Germany). Conjoined electrodes on both ventral upper pelvic spines (H93G, Covidien, Germany) served as the reference or central electrodes, whereas the ground electrode was placed at Th11 level. In addition, we also attached one pair of bipolar electrodes along the heart axis to reliably detect heart activity for subsequent artefact elimination. All analogue signals were simultaneously analogue-to-digital converted at a sampling rate of 2,048/s (Tower of Measurement, DeMeTec, Germany, amplitude resolution: 24 bit at ± 5 V (6 nV/bit), anti-aliasing filter at 1,006 Hz) and stored on computer (ATISArec, GJB, Germany) for further offline analysis.

During data analysis signals were first cleaned from ECG interferences by applying a template-based algorithm [32]. During execution of the endurance task start and stop markers were placed in the measurement file, that were used to define the analysis window. For SEMG analysis signals were band pass filtered between 20 Hz and 250 Hz. To account for possible interferences from the power circuit also a 50 Hz notch filter was applied. Single root mean square (rms) and mean frequency (MF) values were calculated over periods of 1024 samples (0.50 s) moving continuously over the whole record time with 50% overlap (0,25 s).

To analyse rms and MF changes during the endurance task deviations from start level (mean values during first five seconds) were calculated as relative changes. For this analysis, the individual endurance times were normalized to 100%, since not all participants completed the 600 s target time.

### 2.4 Statistical analysis

We used a linear mixed-effects model (LMM) to evaluate the main effects of "group", "side", and "electrode position" together with interactions between these factors for relative rms and MF changes. "Group", "side", and "electrode position" were modelled as fixed effects with a random intercept per subject. Initially, all main effects and interactions were calculated, but for the final analysis, only the significant main effects together with significant interactions remained in the calculation. Significance levels for group differences per electrode position and also the pairwise comparisons between electrode positions per group were adjusted by the least significant difference, together with always providing mean differences plus lower and upper borders of the 95% confidence intervals.

## 3 Results

All endurance-trained participants finished the target time of 600 s. In group CON one participant failed to reach the target time (breakup time 352 s), but in the ST group altogether eight out of 13 participants had to give up prior to target time (breakup times 195 s to 490 s).

### 3.1 Relative rms change

The initial calculation revealed significant main effects of "group" (p = 0.004) and "electrode position" (p<0.001) together with a significant interaction of "group * electrode position" (p<0.001), whereas "side" and the interactions "group * side" and "side * electrode position" showed no systematic effects (p values 0.062 to 1.00).

Electrode position-independent mean relative rms changes over time were +27.87% (95% CI +0.83% to +54.91%) for CON, +14.17% (95% CI -10.70% to +39.04%) for ET, and +73.53% (95% CI -48.66% to +98.41%) for ST and were therefore significant for ET vs. ST (p = 0.002) and CON vs. ST (p = 0.016) but showed no systematic difference for ET vs. CON (p = 0.454).

The calculated pairwise comparisons per electrode position also showed significant differences for ST vs. ET and CON, but not for ET vs. CON. For ET vs. ST 11 out of 16 side-independent electrode positions showed significant differences with always larger relative changes for the ST group (Table 2). The comparisons of CON vs. ST showed significant differences for the two upper electrode rows and position nine, which is the most lateral one of the third row (Table 2). In accordance with the position-independent results, no systematic differences for ET vs. CON could be determined.

**Table 2:** Pairwise comparisons of the relative rms changes between groups per side-independent electrode position (EP).

	ET vs. ST			CON vs. ST			ET vs. CON		
		95% CI			95% CI			95% CI	
	mean diff.	lower border	upper border	mean diff.	lower border	upper border	mean diff.	lower border	upper border
**EP1**	-121.63	-161.76	-81.51	-100.54	-142.45	-58.63	-21.09	-63.00	+20.82
**EP2**	-98.75	-138.88	-58.62	-85.76	-127.67	-43.85	-12.99	-54.90	+28.92
**EP3**	-78.69	-118.81	-38.56	-70.30	-112.21	-28.39	-8.38	-50.29	+33.53
**EP4**	-65.64	-105.76	-25.51	-55.54	-97.45	-13.63	-10.09	-52.00	+31.82
**EP5**	-100.25	-140.37	-60.12	-79.69	-121.60	-37.78	-20.56	-62.47	+21.35
**EP6**	-72.75	-112.87	-32.62	-61.84	-103.75	-19.93	-10.91	-52.82	+31.00
**EP7**	-49.14	-89.26	-9.01	-38.71	-80.62	+3.20	-10.42	-52.33	+31.49
**EP8**	-42.90	-83.02	-2.77	-31.08	-72.99	+10.83	-11.81	-53.72	+30.10
**EP9**	-78.69	-118.82	-38.56	-64.78	-106.69	-22.87	-13.91	-55.82	+28.00
**EP10**	-46.68	-86.81	-6.56	-32.99	-74.90	+8.92	-13.69	-55.60	+28.22
**EP11**	-29.27	-69.40	+10.85	-14.47	-56.38	+27.44	-14.80	-56.71	+27.11
**EP12**	-27.76	-67.99	+12.47	-16.22	-58.23	+25.79	-11.54	-53.45	+30.37
**EP13**	-49.67	-89.79	-9.54	-36.18	-78.09	+5.73	-13.49	-55.40	+28.42
**EP14**	-34.61	-74.73	+5.52	-18.41	-60.32	+23.50	-16.20	-58.11	+25.71
**EP15**	-30.38	-70.50	+9.75	-12.76	-54.67	+29.15	-17.62	-59.53	+24.29
**EP16**	-23.03	-63.16	+17.09	-11.36	-53.27	+30.55	-11.68	-53.59	+30.23

Values are provided as relative differences (i.e. % difference)

negative values: first group < second group

light shaded cells: p<0.05, dark shaded cells: p<0.01 (adjustment for multiple tests: least significant difference)

**Fig 3:**
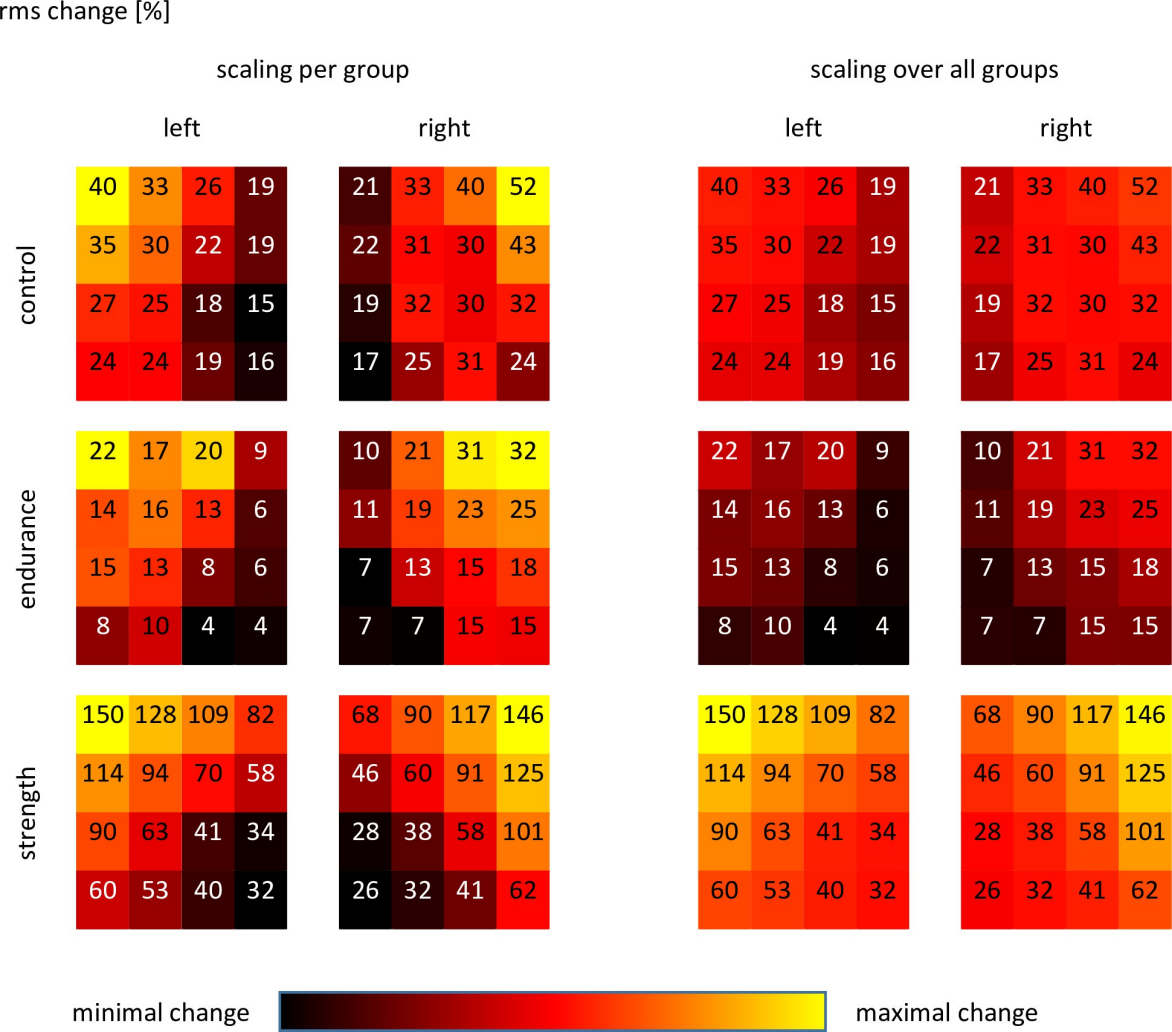
Heat map of the relative rms change for the endurance task. Left side: scaling of relative changes per group. Right side: scaling of relative changes over all groups. Data provided as mean values.

**Fig 4:**
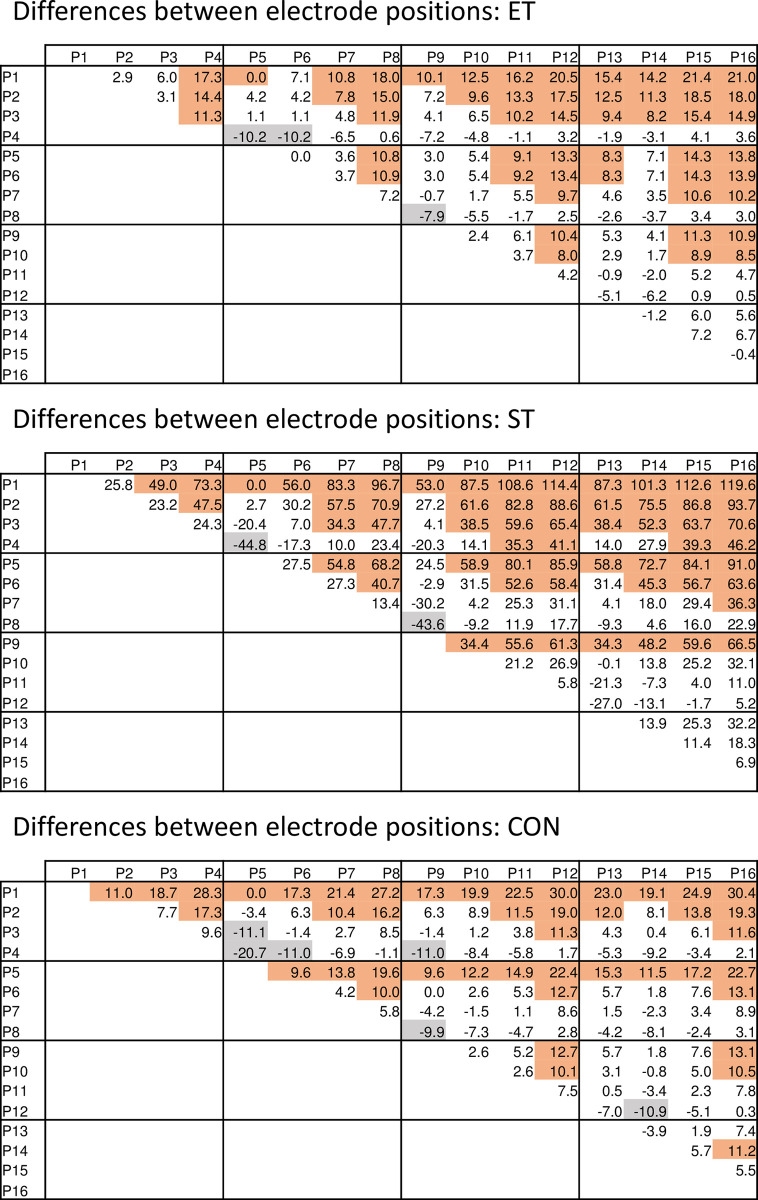
Mean differences between electrode positions for the relative rms change per group. Positive numbers indicate larger values of the positions mentioned in the line header, whereas negative numbers stand for larger values of the positions mentioned in the column header. Shaded cells indicate significant differences (p<0.05; brown for positive values, grey for negative values).

As "electrode position" and the interaction "group * electrode position" were both significant, systematic position-related effects could also be proven which were different for the investigated groups. In Fig 3 a map-like display of the relative rms changes is provided, where values decrease form lateral to medial and cranial to caudal, independent of group. This could be statistically proven independent of group but the number of significant differences between electrode positions varied between groups with the largest number of differences in the ST group, i.e. the most pronounced spatial differences. The relative number of significant differences for the 120 individual comparisons were CON 41.7%, ET 45.0%, and ST 51.7% (for details see Fig 4).

### 3.2 Relative MF change

The initial calculation, including all main effects and their interactions showed significant main effects for "group", "side", and "electrode position" (all p<0.001) with a significant interaction for "group * electrode position" (p = 0.001). The remaining interactions were not significant (p values 0.466 to 0.695).

Electrode position-independent mean relative MF changes over time were and -8.21% (95% CI -12.69% to -3.74%) for CON, -7.65% (95% CI -11.77% to -3.53%) for ET, and -20.09% (95% CI –24.20% to -15.97%) for ST and were therefore significant for ET and CON vs. ST (both p<0.001) but showed no systematic difference for ET vs. CON (p = 0.851).

The pairwise group-comparisons per electrode position showed significant differences for ET and CON vs. ST at all positions (all p values <0.01), whereas ET vs. CON showed no single difference at all (Table 3).

**Table 3:** Pairwise comparisons of the relative MF changes between groups per side-independent electrode position (EP).

	ET vs. ST			CON vs. ST			ET vs. CON		
		95% CI			95% CI			95% CI	
	mean diff.	lower border	upper border	mean diff.	lower border	upper border	mean diff.	lower border	upper border
**EP1**	+11.51	+5.20	+17.82	+9.72	+3.13	+16.31	+1.80	-4.79	+8.39
**EP2**	+11.22	+4.91	+17.53	+9.63	+3.04	+16.22	+1.59	-5.00	+8.18
**EP3**	+12.47	+6.16	+18.78	+11.55	+4.96	+18.14	+0.93	-5.66	+7.52
**EP4**	+14.78	+8.47	+21.09	+15.89	+9.30	+22.48	-1.11	-7.70	+5.48
**EP5**	+10.59	+4.28	+16.90	+9.95	+3.36	+16.54	+0.64	-5.95	+7.23
**EP6**	+8.74	+2.43	+15.05	+8.84	+2.25	+15.43	-0.11	-6.70	+6.48
**EP7**	+10.81	+4.50	+17.12	+10.14	+3.55	+16.73	+0.67	-5.92	+7.26
**EP8**	+13.57	+7.26	+19.88	+15.06	+8.47	+21.65	-1.49	-8.08	+5.10
**EP9**	+10.57	+4.26	+16.88	+11.60	+5.01	+18.19	-1.04	-7.63	+5.55
**EP10**	+10.91	+4.60	+17.22	+11.17	+4.58	+17.76	-0.26	-6.85	+6.33
**EP11**	+13.19	+6.88	+19.50	+11.32	+4.72	+17.90	+1.87	-4.72	+8.46
**EP12**	+11.58	+5.27	+17.89	+12.58	+5.99	+19.17	-1.00	-7.59	+5.59
**EP13**	+11.69	+5.38	+18.00	+12.00	+5.41	+18.59	-0.31	-6.90	+6.28
**EP14**	+13.99	+7.68	+20.30	+12.75	+6.16	+19.34	+1.24	-5.35	+7.83
**EP15**	+17.09	+10.78	+23.40	+13.79	+7.20	+20.38	+3.30	-3.29	+9.89
**EP16**	+16.29	+9.98	+22.60	+13.98	+7.39	+20.57	+2.31	-4.28	+8.90

Values are provided as relative differences (i.e. % difference)

negative values: first group < second group

shaded cells: p<0.01 (adjustment for multiple tests: least significant difference)

For the significant main effect "side" electrode position-independent MF changes values were always larger for the right side (left side: -11.17%, 95% CI -13.63% to -8.71%, right side: -12.80%, 95% CI -15.26% to -10.34%). As no significant interaction between "side" and "electrode position" could be proven, all position-related comparisons were preformed independent of side.

**Fig 5:**
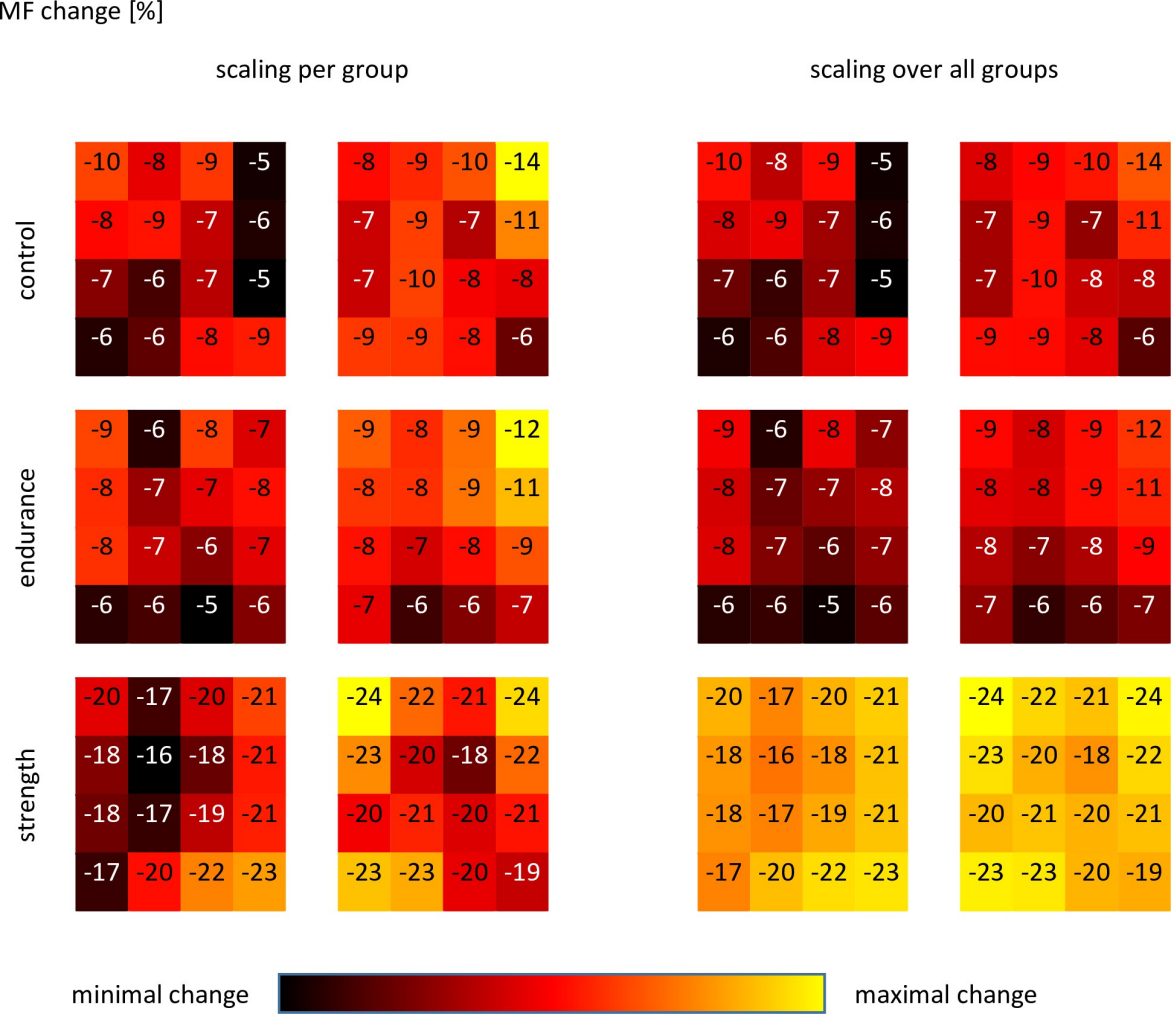
Heat map of the relative MF change for the endurance task. Left side: scaling of relative changes per group. Right side: scaling of relative changes over all groups. Data provided as mean values.

**Fig 6:**
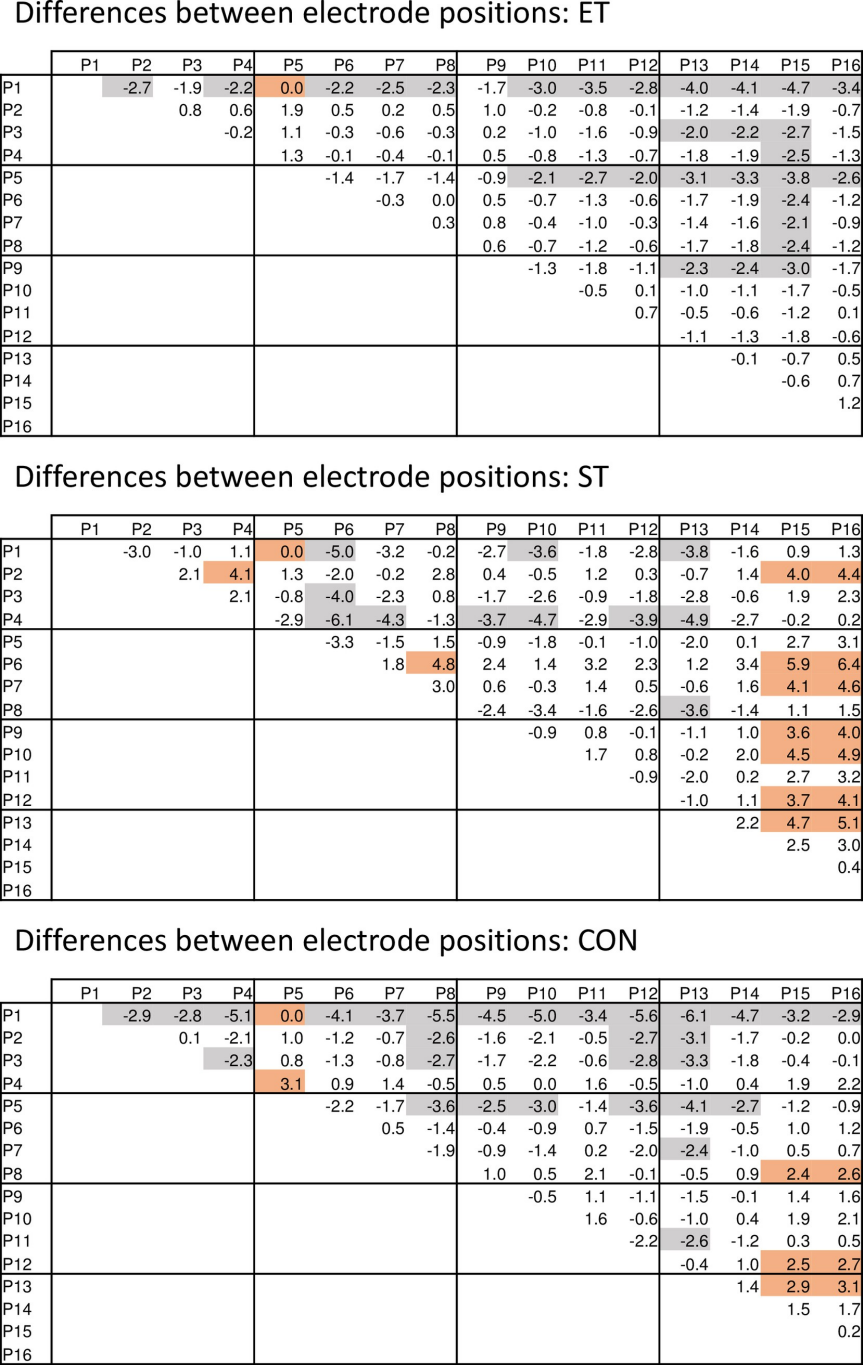
Mean differences between electrode positions for the relative MF change per group. Positive numbers indicate larger values of the positions mentioned in the line header, whereas negative numbers stand for larger values of the positions mentioned in the column header. Shaded cells indicate significant differences (p<0.05; brown for positive values, grey for negative values).

Similar to the position-related differences for the rms changes, also relative MF changes were different between electrode positions, and were further subject to group. In Fig 5 a map-like presentation of the relative MF changes per electrode position is provided, whereas in Fig 6 pairwise comparisons between the single positions are provided. The relative number of significant differences was 30.8% for CON, 25.0% for ET, and 23.3% for ST.

## 4 Discussion

During a ten minutes back muscle endurance task both, relative amplitude and frequency changes varied between groups of differently active and sedentary healthy male subjects and, showed a clear spatial distribution pattern for the observed amplitude changes. To specifically address the question of potential regional differences of fatigue-induced amplitude and frequency changes, we applied a monopolar montage as this particular application is best suited to analyse SEMG signals with respect to spatial characteristics [33].

This endurance task was performed to evoke fatigue in the back muscles to analyse fatigue-related changes of the stressed muscles between differently trained subjects. By definition fatigue is "any exercise induced reduction in the ability of a muscle to generate force or power”" [2], that is accompanied by typical changes of SEMG signal properties. These changes involve an increase of amplitude together with a decrease of the frequency content [34] and were put into an assessment scheme by Luttmann and colleagues in 1996 [35]. Amplitude elevations during fatigue can occur even if the respective firing rate is reduced, or motor units are out of sync. Also, a lengthening of intracellular action potentials bears the potential to increase SEMG amplitude values during fatigue [36]. For the actual investigation the observed amplitude changes may be attributed to recruitment of additional high-threshold motor units to compensate for the occurring temporal loss of active motor units due to energy depletion [37]. This energy depletion, on the other hand leads to a reduction of the conduction velocity of the evoked action potentials [38, 39], which in combination with additional factors like a higher degree of synchronisation between the respective motor units [40, 41] as motor units tend to fire at similar rates [2, 42, 43] results in a reduction of the frequency content of the respective SEMG signal.

With respect to the cranial to caudal orientation the observed relative rms change was largest at the most cranial positions, i.e. the thoracic-lumbar transition which is in good agreement with already published data during submaximal isometric back muscle activation [44]. This finding is also supported by histochemical data for fibre type identification that could prove a larger cross sectional area of both, type I and type II fibres in the thoracic parts of the erector spinae muscle (mean fiber area: 6,241 μm^2^; [30, 45], whereas more caudal fibres were lower in diameter (mean fibre area: 4,897 μm^2^ [30]) but showing a larger proportion of type I fibres (cross sectional area ratio I/II thoracic: 1.024, lumbar: 1.085, [30]). As type I fibres are less prone to fatigue these results are in good agreement with the results of our study.

With respect to the lateral to medial orientation of the relative rms change, the more laterally located electrodes showed larger relative changes in comparison with the more medial located electrodes, i.e. those located closer to the vertebral column. For the lateral to medial differences one has to consider that other back muscles have been measured with the applied electrode grid as well. This particularly includes the iliocostalis and the latissimus dorsi muscles. Both muscles are considered as mobilizing muscles that are not suited to provide stability, i.e. prolonged static activation [46]. In summary, the systematic spatial differences found in the evoked rms changes are comprehensible.

Similar considerations account for the found group differences, that were most pronounced between the strength-trained participants and the endurance-trained subjects together with the sedentary controls: Relative amplitude changes were found to be largest in the group of strength-trained subjects, followed by physically inactive healthy controls. Least relative amplitude change levels were observed in the group of endurance trained subjects. Although, the strength-trained participants showed largest maximum force values of their back muscles (Anders and Schönau submitted) and therefore had the most advanced physiological power reserve, eight out of 13 strength trained subjects failed to complete the ten minutes endurance task. As all active participants trained at competition level, especially for the strength trained subjects considerable increased type I and especially type II cross sectional area of their back muscles can be assumed [47, 48]. This, at a group-specific level again alters muscle blood supply during prolonged static contraction, causing elevated muscular fatigue. Since all active participants trained at competition level they were all highly motivated, excluding motivational reasons for the premature surrender in eight of 13 participants with high certainty.

In contrast to the amplitude changes, fatigue-related frequency alterations were not systematically distributed across the investigated region, but showed a slight, but significantly more pronounced change on the subject’s right side. Again, group had a strong impact on relative MF change. The observed systematic side difference may be explained by the fact that except two subjects (one in group ET, one in group CON) participants were right-handed. The preferred use of the dominant side requires different adjustments depending on the observed region. All agonistic muscles will be trained and therefore are expected to be more fatigue resistant [49]. In contrast, in the back region, due to contralateral stabilization the non-dominant region was found to be more fatigue resistant [50]. Similar to the fatigue related changes of rms values the MF change was again largest for the strength trained group. As already stated, the assumable large fibre diameters for the ST group may lead to deficits in blood and therefore metabolic supply and waste removal.

## 5 Limitations

To account for individual anthropometry we used grids with different edge lengths. This, by adhering to the subject’s proportions precluded bipolar calculations. Therefore, monopolar signals were analysed. These signals by nature contain crosstalk from other than back muscles, especially from the latissimus dorsi and iliocostalis muscles for the lateral and cranial electrodes as the reference electrode was located ventral. This also affects reliable frequency change analysis and may at least in part explain the less systematic results of relative MF changes. On the other hand, this adopted grid size enabled a close correlation to spinal vertebral levels as the edge length was defined according to the distance between L1 and L4 palpable spinal processes. Therefore, the cranio-caudal orientation was strictly defined according to morphological landmarks.

Not all participants completed the requested endurance time of 600 s. One participant of the sedentary group, but overall eight of the strength-trained group failed. To compensate for the dropouts we therefore analysed time-normalized data. In other words, we analysed endurance data of eight out of 13 strength- trained participants who performed the endurance task until exhaustion. This was not the case for the endurance- trained group and accounted for only one participant of the sedentary control group. It is therefore not clear which rms and MF change values would have been determined if all participants had performed the static endurance test until exhaustion. This must be taken into account when interpreting the results.

Surface EMG recordings with significant loads over such long periods of time bear the problem of sweating of the subjects, which can lead to lever off the electrodes and may also alter the contact resistance between the skin and the electrodes. The first issue in particular must be controlled and remedied if possible, whereas resistance changes are difficult to control but may affect the observed amplitude changes.

## 6 Conclusions

In this study, the static endurance capacity of back muscles at 50% UBW was investigated in healthy young male subjects. It turned out that the majority of strength-trained participants failed to complete the requested ten minutes endurance time. Although, especially in the light of an aging population strength training has its merits our results question the functional suitability of frequent and isolated high-impact strength training to also compensate for everyday endurance requirements (like doing the dishes), as already sedentary healthy male subjects showed functionally superior results during fatigue. Relative amplitude changes during the endurance task were characterized by a systematic spatial distribution pattern, showing largest elevations at the most cranial and lateral electrode positions. Electromyographic signs of fatigue were most elevated in the strength trained group, but may be altered by their predominantly exhaustive task execution.

## Supporting information

S1 Text Activity level definition. (DOCX)

S1 Data Study data. (XLSX)
