# Supplementary material for: Spatiotemporal characteristics of lower back muscle fatigue during a ten minutes endurance test at 50% upper body weight in healthy inactive, endurance, and strength trained subjects
Source: PLoS One. 2022 Sep 13;17(9):e0273856. doi: 10.1371/journal.pone.0273856 (PMC9469946; doi:10.1371/journal.pone.0273856)
Supplement: S1 Text — (DOCX) [file pone.0273856.s001.docx]

Activity level definition

| A 1 | No physical activity at all |
| --- | --- |
| A 2 | Low physical activity. Few and light physical activities (walking, light gardening, etc.). |
| A 3 | Medium physical activity. Physical activity, at least two to max. four times per week, 30 minutes each. |
| A 4 | High physical activity. Physical activity, at least four to max. six times per week, 30 minutes each. |
| A 5 | Very high physical activity. Intense physical activity, intense training or hard physical work, daily, at least one hour each time. |
